# Strength is in engagement: The rise of an online scientific community during the COVID‐19 pandemic

**DOI:** 10.15252/embr.202152612

**Published:** 2021-05-05

**Authors:** Christine E Cucinotta, Benjamin J E Martin, Melvin Noé González, Pravrutha Raman, Vladimir B Teif, Hanneke Vlaming

**Affiliations:** ^1^ Division of Basic Sciences Fred Hutchinson Cancer Research Center Seattle WA USA; ^2^ Department of Biological Chemistry and Molecular Pharmacology Blavatnik Institute Harvard Medical School Boston MA USA; ^3^ Department of Cellular and Molecular Medicine University of Copenhagen Copenhagen Denmark; ^4^ School of Life Sciences University of Essex Colchester UK

**Keywords:** S&S: Careers & Training, Methods & Resources, S&S: Ethics

## Abstract

Many scientists, confined to home office by COVID‐19, have been gathering in online communities, which could become viable alternatives to physical meetings and conferences.

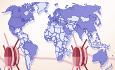

As COVID‐19 has brought work and travel to a grinding halt, scientists explored new ways to connect with each other. For the gene regulation community, this started with a Tweet that quickly expanded into the “Fragile Nucleosome” online forum, a popular seminar series, and many intimate discussions connecting scientists all over the world. More than 2,500 people from over 45 countries have attended our seminars so far and our forum currently has ~ 1,000 members who have kick‐started discussion groups and mentorship opportunities. Here we discuss our experience with setting up the Fragile Nucleosome seminars and online discussion forum, and present the tools to enable others to do the same.

Too often, we forget the importance of social interactions in science. Indeed, many creative ideas originated from impromptu and fortuitous encounters with peers, in passing, over lunch, or during a conference coffee break. Now, the ongoing COVID‐19 crisis means prolonged isolation, odd working hours, and less social interactions for most scientists confined to home. This motivated us to create the “Fragile Nucleosome” virtual community for our colleagues in the chromatin and gene regulation field.

… the ongoing COVID‐19 crisis means prolonged isolation, odd working hours and less social interactions for most scientists confined to home.

While the need to address the void created by the COVID‐19 pandemic triggered our actions, a large part of the international community already has had limited access to research networks in our field. Our initiative offered new opportunities though, in particular for those who have not benefited from extensive networks, showing how virtual communities can address disparities in accessibility. This should not be a stop‐gap measure during the pandemic: Once we come out from our isolation, we still need to address the drawbacks of in‐person scientific conferences/seminars, such as economic disparities, travel inaccessibility, and overlapping family responsibilities (Sarabipour, 2020). Our virtual community offers some solutions to the standing challenges (Levine & Rathmell, 2020), and we hope our commentary can help start conversations about the advantages of virtual communities in a post‐pandemic world.

… once we come out from our isolation we still need to address the drawbacks of in‐person scientific conferences/seminars, such as economic disparities, travel inaccessibility and overlapping family responsibilities…

## Finding like‐minds through Discord

On March 19 last year, Christine enquired on Twitter about scientists’ interest in participating in a forum dedicated to discussing chromatin and transcription. The same day, she founded our community on Discord, a free online platform (https://discord.gg/dXqT89r). She named it “Fragile Nucleosome”, since “it can be here for us when we need it and it can go away when we don’t” (https://twitter.com/chrstn_e/status/1240696318093189126?s=20). The six of us—five postdocs and one principal investigator from different countries—represent the initial committee created for running both the Fragile Nucleosome Discord server (hereafter FN server) and the seminar series in 2020. To date, we have attracted ~ 1,000 members to the FN server. We have a steady group of ~ 300 active members, connected through various discussions and events. On a Discord server, administrators can create topics that contain subcategories, or “channels”, where members can post messages. The FN server has about 25 different channels for discussions, including scientific topics on chromatin, transcription, methods, and more general topics such as career development and events (see Box 1).

Box 1 **Technical aspects of running the Fragile Nucleosome server.**

**Discord vs Slack**

Most scientific virtual communities have been formed on Slack and Discord. Slack has the advantage of being widely used in laboratories already, and it allows “threads” or branched conversations. The Discord platform has mostly been used by gamer communities. In contrast to Slack, the free version of Discord stores old messages, generally offers the admins more options, and has many different “bots” to automate certain tasks. Different reasons may weigh more heavily for different communities, and there may be other suitable platforms that we have not looked into. Here we share some tips & tricks to set up a Discord server for your virtual community.

**Template server & getting started**

Based on our Fragile Nucleosome server, we have created a template Discord server: https://discord.new/6tKexD5qz6jN with member roles, categories and channels set up as described below. For more information on setting up a Discord server, see https://support.discord.com/hc/en‐us/categories/200404378‐Server‐Setup.

**Member roles and new member onboarding**

To help people to find and join the server, you will want to make it public. The downside is that people with ill intentions could join to spam your channels. To prevent this, we assign roles with different sets of permissions. This has also been useful as different groups of volunteers organized their own activities.

Under Server Settings, you can create new roles and define default permissions for each role. New people joining the server are first in the category @everyone and will have read‐only permission; you could also keep most channels hidden from @everyone. We created a “member” role, which has both read and write access, and an admin role which has permissions to delete messages, reorganize channels, change member roles etc.

We created a #joining channel where @everyone does have writing permission and where the admins ask new members to identify themselves by name and affiliation, after which we will upgrade them to the member role. We’ve had one occasion of spam in this channel, but full members don’t see this channel, therefore it has not been disruptive to the community.

**Categories and channels**

We found that making too many specialized subchannels leads to many channels with little discussion each and makes the server overwhelming to new members. At the time of writing this manuscript, we limited our science category to 6 channels, which should be defined broadly enough to cover the whole chromatin and transcription field. On top of that, we have a category to discuss methods. Other categories are General, Events and Career, as well as New members & Help. Finally, we’ve created two categories that are invisible to most: a Behind‐the‐scenes category for organization purposes and an Archive category with channels that we culled.

**Invites**

Be careful with invites as some expire after 24 h, and it may frustrate people to receive a non‐functional link. An invite will always link to a specific channel, so if you set up something akin to our #joining channel, make sure your invite links there.

**Discord Bots**

The most important bot we have installed is Dyno (https://dyno.gg) to help manage the server. We use the Action log module to let Dyno write messages in our #hidden‐joininglog channel when new members join. We use the Announcement module to send new members a direct message to explain the onboarding procedure. Finally, Dyno is helpful to easily clean up messages in the joining channel.

Another bot we’ve added is Simple Poll (https://simplepoll.rho.sh), to run polls on the server, which makes voting as easy as clicking an emoji.

Statbot keeps track of server activity and makes plots over time, for instance, what channels are most used, how many members are online on specific days, etc.

The Sesh bot can be used to maintain an #events‐calendar that can include event information and registration links, and that lets people RSVP, set reminders, display times in their own time zone, et cetera.

There are many successful online communities. For example, the Slack servers NewPI (https://newpislack.wordpress.com/), FuturePI (https://futurepislack.wordpress.com/), and UK_NewPI (https://uknewpi.slack.com/) are focused on career development and connecting researchers at similar career stages (Acton *et al,* 2019). The Fragile Nucleosome community is instead focused on a scientific field: It offers experts and newcomers alike a place to discuss science and provides multiple avenues to connect with other scientists. The FN has grown into an inclusive and international community, offering mutual help and support.

The success of any online community depends on the engagement of its members, and we have been fortunate with our community’s participation. The team of six behind the seminar series (with Matteo Perino instead of Melvin Noe Gonzalez in 2021) handle most of the administrative tasks, and we engage members on the FN server by actively seeking their feedback on ideas and plans, and welcoming new initiatives. The diversity in laboratories and background of the participants has provided many different viewpoints and exciting discussions. For example, a group of trainees spearheaded an innovative journal club where they invite an expert in the field to discuss a preprint or peer‐reviewed article online (https://generegulation.org/fragile‐nucleosome/fn‐journal‐club/). Other initiatives include recurring social events, a writing group, and a monthly diversity, equity, and inclusion (DEI) discussion group on racial disparities in science and how to effectively implement change. Similar discussions in our community sparked the idea to initiate a global mentoring program. We were amazed by the number of mentees and mentors signing up and were able to establish 42 mentoring pairs.

The diversity in laboratories and background of the participants has provided many different viewpoints and exciting discussions.

## Science on‐demand: virtual seminars at home

Less than a week after we started the FN server, Hiten Madhani from UCSF volunteered to give the first talk on April 1, 2020. We quickly put together a Zoom webinar and, despite the short notice, almost a hundred people attended. This was the start of a weekly seminar series (https://generegulation.org/fragile‐nucleosome/). The virtual format was new to the speakers, the attendees, and the organizers. However, we quickly came to appreciate the strengths of online platforms. We heard from the speakers how much they liked the convenience of presenting from home or work, avoiding the travel burden and large carbon footprint associated with in‐person seminars. Online meetings also offer scientists with limited time to travel great opportunities to present their work to their peers (“Online meetings for the win”, 2020; Sarabipour, 2020). Many of our attendees are from institutes or countries rarely frequented by well‐established scientists; our seminars provide an easy and affordable opportunity for them to hear and interact with senior scientists. This sentiment was captured during the Q&A of Karolin Luger’s seminar when a graduate student from India said, “I can’t believe I’m asking you a question!”.

**Figure 1:**
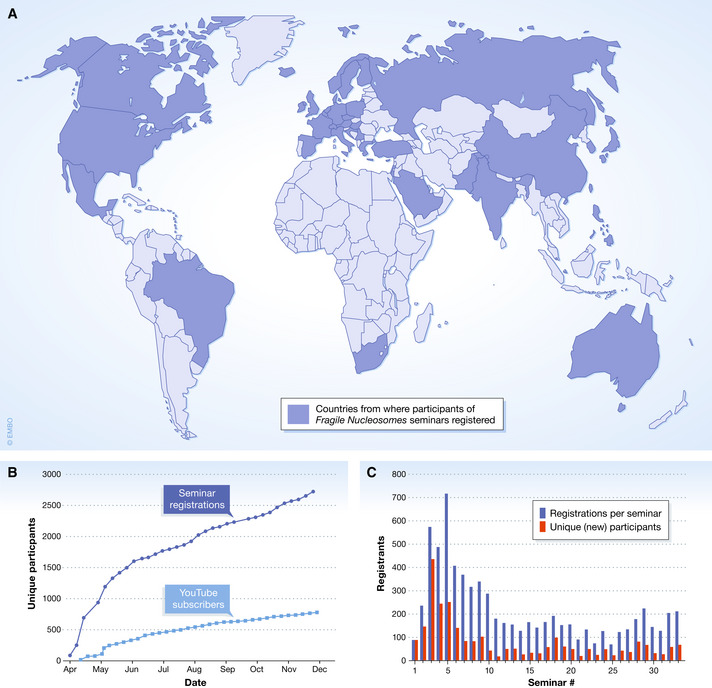
Fragile Nucleosome seminar series participation (A) Participants of Fragile Nucleosome seminars have registered from 45 countries from all continents. (B) The cumulative number of scientists registered for our seminars (black line) and YouTube subscribers (blue). (C) The numbers of total (pink) and unique registrations per seminar (gray). Numbers are based on participant’s registration for the Zoom seminars. The map was created using the Interactive Maps plugin for WordPress by Freemius (freemius.com). The statistics for the Fragile Nucleosome YouTube channel (Fig 1B) were collected using YouTube Studio. The audience growth data was visualized with Origin Pro 2020 (originlab.com).

Now, one year into the Fragile Nucleosome series, we have hosted more than 60 speakers. The talks have been of excellent quality and covered research on chromatin and transcription with a wide range of sub‐topics, approaches, and organisms. Since the first seminar, we have had more than 2,500 unique scientists registered as participants from 45 countries from all continents (Fig 1A); however, the increase of new seminar participants has not yet saturated (Fig 1B). In addition to a core group who attend most sessions, 10‐40% of seminar participants are first‐timers (Fig 1C), which shows that we continue to attract new scientists. While our seminars were most highly attended during the strict lockdown in April/May 2020, we have kept a consistent audience (Fig 1C). Most talks are available on YouTube with speakers’ permission to give those who may have otherwise missed the seminar an opportunity to watch them at a convenient time.

Many of our attendees are from institutes or countries rarely frequented by well‐established scientists; our seminars provide an easy and affordable opportunity for them to hear and interact with senior scientists.

Our YouTube audience consists of almost 900 subscribers and also continues to increase. Recording talks has been especially helpful for those with family demands or living in time zones incompatible with our seminar time. Several members have used these recordings in courses they teach.

As the scientific community came to grips with the sudden cancelation of physical seminars and conferences, we approached senior scientists and invited them to present in our online initiative and many of them enthusiastically agreed. While this first wave of seminars was critical to our rapid growth, we noticed that the same speakers we had intended to invite, or had invited already, and were also being scheduled in other online seminar series. Here, we saw the perfect opportunity to diversify our speaker lineup. We split our one‐hour seminar into two talks: one by an independent scientist and one by a trainee. The speakers were from different laboratories, but we aimed to pair them based on a common topic when possible. We also hosted a “meet‐the‐speaker” session where seminar participants could interact with speakers after their talk.

To improve diversity, we have regularly used an online form where scientists could volunteer to give a seminar no matter their career stage. This allowed us to host researchers outside of our immediate network, often early‐career scientists and from different geographical locations and institutes than our own. However, this approach still requires careful attention to balance self‐volunteered candidates with encouraged nominations and invitations to maintain gender balance, and representation from groups that are too often underrepresented in our field. Through this continuous effort, we put together a lineup of speakers balanced in gender and who range from junior to senior scientists. Our 2020 speaker lineup was 49/51% female/male and 42/25/33% early‐career/assistant or associate prof/full professor, respectively.

Finally, the post‐seminar speaker meetings allow scientists of all career stages and from all over the world to ask questions and seek advice on career trajectories. While such informal speaker meetings are common at many institutes, they can be limited by departmental budgets, creating another barrier for those at less privileged institutes.

## Lessons learned and future plans

When we created the Discord server, we wanted a dedicated platform for people to meet other researchers with similar interests and to share questions, interesting papers, and the occasional meme. However, as any online community sooner or later discovers, one of the never‐ending challenges is keeping members engaged and active. As described above, seeking members’ feedback about events and server administration has instilled a sense of community and engagement. A caveat for an open event such as our seminar series is choosing a seminar time that is convenient for everyone. We attempted to tackle this problem in a couple of ways. First, we offer speakers the option to change their seminar time. On one occasion, we changed our time to better suit European and Asian countries, which helped increase attendance from these parts of the world. Second, we encourage speakers to record their seminars, which allows attendees to watch it at a more convenient time. While this has been a popular choice, some speakers are skeptical of this option—particularly when they present unpublished data. Some alternatives we offer are making the talk available for a limited period of time or at a later date, or to edit and upload only parts of the talk that describe published data.

However, other problems have been more challenging to resolve. An issue with the new virtual format has been to recapitulate the interactive and networking opportunities of in‐person seminars. To address this, our seminars are followed by “face‐to‐face” zoom meetings between speakers and interested participants. However, this has had variable success. We suspect that differences in time zones, zoom fatigue, and pandemic responsibilities may prevent many from spending extra time to chat with speakers. We are now trying different iterations of these meetings to encourage more participation. Another challenge our speakers have faced is simply due to the nature of online seminars: the lack of real‐time feedback from an audience. Indeed, a live audience provides features a speaker can fixate on while presenting—a reassuring nod, an attentive gaze, a dangerous yawn—which are all lacking in a webinar. While we are trying to think of creative ways to solve this, this feeling of “talking to the void” may be one of the many things we may have to adapt to in this new reality.

The question then is, where do we go from here? The pandemic exposed shortcomings of the classical scientific conferences and seminars. First, while many institutes hold weekly seminar series, not all institutes have the resources to host prominent speakers. Second, while multi‐day conferences often provide a forum to listen to and interact with leaders in the field, they are a privilege reserved for those without financial strain, family commitments, or immigration restrictions. Talented scientists in developing countries often miss out on interactions with peers and world leaders in their field. It is now up to us, in privileged positions to notice, acknowledge, and act.

From our constant experimenting with different projects, we have developed a template to start and run scientific communities such as ours (See Box 1) and surmise four key aspects that can provide a starting point to improve inclusivity in academia.

1. **A dedicated online platform where researchers at all levels can interact with each other.** To attain a sense of community, there has to be constant, real interactions between those involved. The Fragile Nucleosome Discord server is one such space where people can interact, ask questions, and even have the chance to find new collaborators. We strongly believe similar groups could be ultimately helpful to other scientific fields.
2. **Virtual seminar series.** Having an online series can keep a scientific community well‐connected with the latest findings in a field. This also has expanded scientific networks and started new collaborations. Additionally, we can give young scientists a platform to show their work and become known in the field. Regular seminars increase the number of speakers we can spotlight, and it creates a ritual of listening to science each week with your peers worldwide.
3. **Recording of talks.** Whether due to family responsibilities or time zones, many people are unable to attend every week. We therefore record talks with permission from the speakers and make them publicly available on our YouTube channel. To encourage recording talks, an intriguing future possibility is to allow talks to be cited in future papers, similar to preprints.
4. **A physical‐online seminar/conference hybrid.** There is a social benefit of meeting old peers and new ones at conferences. However, there is more to gain by including live streaming. This would increase the number of attendees that can be exposed to cutting‐edge science and, with technological improvements, give them a chance to present posters virtually. Furthermore, such online seminars provide opportunities for trainees around the world to both present their data and interact with speakers online.

Given our experience with the Fragile Nucleosome Discord server, we encourage those in other disciplines to create communities of their own. To this end, we have created an open‐access template (see Box 1) that anyone can use to jump‐start their own community. Indeed, we would be remiss if we did not acknowledge that those of us involved in these projects are privileged enough to have an abundance of time during the lockdown, giving us the chance to volunteer and bring together this community. However, as most of us return to working in the laboratory, we anticipate having to recruit more interested people to split our workload. While we cannot entirely replace in‐person meetings, online symposiums held a few times a year and virtual platforms can serve as additional means to find new collaborators, colleagues, and friends. It has truly been a joy to watch this online space grow, and for this, we are extremely grateful to all the members of our Fragile Nucleosome community.

While we cannot entirely replace in‐person meetings, online symposiums held a few times a year and virtual platforms can serve as additional means to find new collaborators, colleagues and friends.

## Conflict of interest

The authors declare that they have no conflict of interest.
